# Capillariid diversity in archaeological material from the New and the Old World: clustering and artificial intelligence approaches

**DOI:** 10.1186/s13071-025-06715-0

**Published:** 2025-03-12

**Authors:** Victor Hugo Borba, Ludmila Gurjão, Coralie Martin, Benjamin Dufour, Matthieu Le Bailly, Alena Mayo Iñiguez

**Affiliations:** 1 Laboratorio de Parasitologia Integrativa e Paleoparasitologia, Instituto Oswaldo Cruz, IOC-FIOCRUZ, Rio de Janeiro, RJ Brazil; 2 Laboratório de Helmintologia Romero Lascasas Porto, Faculdade de Ciências Médicas, UERJ, Rio de Janeiro, RJ Brazil; 3 Unité Molécules de Communication et Adaptation des Microorganismes (MCAM, UMR 7245), Sorbonne Universités, Muséum National d’Histoire Naturelle, CNRS, CP52 Paris, France; 4 Université Marie et Louis Pasteur, CNRS UMR 6249 Chrono-environment, Besançon, France

**Keywords:** Paleoparasitology, Capillariidae, Archaeology, Parasites, Taxonomy

## Abstract

**Background:**

Capillariid nematode eggs have been reported in archaeological material in both the New and the Old World, mainly in Europe and South America. They have been found in various types of samples, as coprolites, sediments from latrines, pits, or burial. Modern parasitological records show that around 300 species of capillariids have been described in all vertebrate taxa, including humans, making it a very diversified group. The main proposal of this work is to characterize and identify capillariid eggs found in archaeological sites from Europe and Brazil.

**Methods:**

A total of 39 samples of archeological sites from Europe, deposited in the paleoparasitological collection of the University Marie & Louis Pasteur, Besançon, France was analyzed. In addition, 80 coprolites from the pre-Colombian archaeological site *Gruta do Gentio II*, Brazil, deposited in the Paleogenetic Laboratory at Oswaldo Cruz Institute, Oswaldo Cruz Foundation, Rio de Janeiro, were evaluated. Samples were treated according to the protocols of each laboratory and then analyzed under light microscopy. Capillariid eggs were classified according to length, width, plugs, and eggshell sizes, and statistical analysis of the morphometric dataset was performed. Using a reference dataset of specimens provided by both Institutional Collections, three approaches to species identification were applied: discriminant analysis, hierarchical clustering, and artificial intelligence/machine learning.

**Results:**

A total of 10 samples from Europe and 4 from Brazil were positive for capillariid eggs, showing 13 different morphotypes. As European samples were mainly collected from latrines and pits, parasite–host information was absent, and consequently, species identification was impaired. In contrast, the availability of host information rendered the identification of capillariid species for the Brazilian coprolites. The new methodology indicates capillariid species identified on various samples, resulting in the presence of *Capillaria exigua* (Dujardin, 1845) in feline coprolite, *Baruscapillaria resecta* (Dujardin, 1845) in opossum, and *Aonchotheca bovis* (Schnyder, 1906) in bovid, in the Brazilian site, while in European sites, *Capillaria venusta* (Freitas e Mendonça, 1958), *Aonchotheca myoxinitelae* (Diesing, 1851), *Eucoleus madjerdae* (Bernard, 1964), and *Baruscapillaria spiculata* (Freitas, 1933) were found.

**Conclusions:**

The study provides new results by applying innovative methodologies for parasite identification and gaining insights into the past host (human or animal)/parasite relationships.

**Graphical Abstract:**

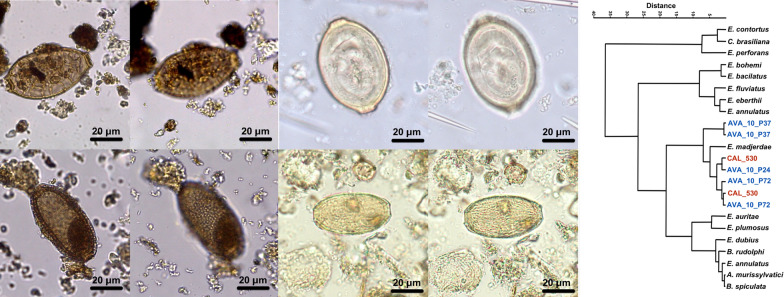

## Background

Paleoparasitology, the study of parasites in archaeological and paleontological material, aims to explain the host/parasite/environment interactions in the past and to elucidate parasite origin and evolution [1]. Parasite eggs, due to their chitinous eggshell, which enables egg preservation through time, are the main structures recovered from ancient samples. Species identification of parasites in paleoparasitological records is one of the obstacles in the field, as it is based solely on egg structures. In some cases, identification at the species level, or even at the genus level, is impossible using accessible scientific literature, which does not always include clear information on the egg structure.

The family Capillariidae (Railliet, 1915) has a very complex and controversial taxonomy, with more than 20 genera described [2, 3]. Capillariids parasitize all vertebrates, such as fish, amphibians, reptiles, birds, and mammals, in various infection sites. Due to this diversity, the identification of parasites in ancient samples has been impaired [4].

In the Old World, archaeological sites often provide Capillariid eggs, but most of the paleoparasitological findings correspond to hollow structures, such as latrines or pits, and have no host definition [5, 6]. In the archaeological site located in the Place d'Armes of Namur, Belgium, capillariid eggs were found in latrines dated to the Gallo-Roman Period (second and third centuries AD), the Carolingian Period (IX–XI AD), and between the twelfth and thirteenth centuries [7]. In some other cases, the origin of the samples is known, for example, in Bouchet [8], who analyzed 23 human coprolites in France, dating to 5150–4930 before present (BP), and found 21 positives for capillariid eggs. In Germany, eggs of capillariids were also found by the analysis of sediment removed from the pelvic region of a skeleton dating to 4500 years [9]. In the Old World, there are still reports of capillariid eggs in archaeological sites of regions that today belong to the countries of France [6], Italy [10], Russia [11], and Czechia [12].

In the New World, most of the findings are in the region of Patagonia, Argentina [5]. Fugassa and Guichón [13] found capillariids eggs in human coprolite of 6540 ± 110 years BP. They were also found in sediments associated with pelvic bone from an archaeological site from the south of the province of Santa Cruz, Argentina [14]. In pellets from the Cerro Casa de Piedra archaeological site, also in Santa Cruz, numerous eggs were identified as *Capillaria* sp. and later as *Calodium hepaticum* (Bancroft, 1893) [15]. However, despite numerous findings of capillariids in South America, there are only two reports of the parasite in Brazil [16, 17]. The scarcity of Capillariidae egg findings in paleoparasitological material does not necessarily indicate the absence of this parasite in the environment or even of infections [18]. When the preservation of archaeological material is very damaged and consequently the morphology of eggs as well, it is difficult to distinguish between trichurid and capillariid eggs by light microscopy [17].

The main proposal of this study is to characterize Capillariidae eggs from archaeological sites in Europe and Brazil and to provide taxonomic information on capillariid eggs diversity, using a reference dataset of specimens from Institutional Helminthological Collections combined with discriminant analysis, hierarchical clustering, and artificial intelligence/machine learning approaches.

## Methods

### Archaeological sites

#### Gruta do Gentio II (GGII)

The site is located in Unaí, Minas Gerais state, Brazil, dated 12,000–3500 BP, with two cultural periods, hunter-gatherer (12,000–7295 ± 150 BP) and horticulturist (3490 ± 120 – 410 ± 60 BP). The cave, where human, animal, and plant remains were found, has an internal area of 200 m^2^ associated with a calcareous wall. A total of 80 coprolites of different shapes were collected [19, 20] and their producers were identified by DNA barcoding analysis [20, 21].

##### La Rochelle Augustin (LRA)

The site is located in western France near the Bay of Biscay and is dated from the seventeenth–eighteenth centuries. The structure studied corresponds to four latrines in which four sediment samples were analyzed under microscopy.

##### Calais ZAC de la Turquerie (CAL)

The site is in northern France. It is dated to the Carolingian Period between the eighth and the tenth centuries, and is located in an old marsh. It has evidence of agropastoral activities, focused on livestock and processing. It was discovered with fauna remains but no evidence of housing, only places with pits; 12 coprolites were analyzed under microscopy.

##### Bourges Avaricum (AVA)

The site is located in the center of France. It corresponds to a craft district built during the eleventh and twelfth centuries and occupied until the seventeenth century. The pits studied are dated between the thirteenth and seventeenth centuries and are linked for the most part to a tannery activity. In this site, 73 samples of organic sediments were studied.

### Paleoparasitological analysis

Two techniques were used for the recovery of parasite eggs according to each laboratory protocol. Brazilian samples were previously identified as GGII-01, *Panthera onca* (Jaguar); GGII-15, *Didelphis albiventris* (white-eared opossum); and GGII-33 and GGII-51, *Bos taurus* (cattle) [20, 21]. They were rehydrated, as suggested by Callen and Cameron [22], with a 0.5% trisodium phosphate solution (Na3PO4.H2O) for 72 h at 4 °C. Then the samples were homogenized and sedimented for 24 h with a triple-folded gauze as proposed by Lutz [23]. A total of 200 microliters of sediment were analyzed for each sample, distributed on 20 temporary slides with glycerol. Samples were examined and analyzed using a Nikon Eclipse E200 microscope at the magnification of 100× and 400× with the software Image Pro Plus—Media Cybernetics, USA. All the processes were carried out at the Paleogenetic Laboratory of the Integrative Parasitology and Paleoparasitology Laboratory (LPIP) from the Oswaldo Cruz Institute (IOC) at Oswaldo Cruz Foundation (FIOCRUZ), Brazil.

European samples were also rehydrated with a 0.5% trisodium phosphate and 5% glycerinated water solution for 7 days and with a drop of formalin solution as well. After the homogenization, the samples are submitted to an ultrasound treatment (50/60 Hz) for 1 min and strained through 315 μm, 160 μm, 50 μm, and 25 μm meshes [6]. A total of six slides were analyzed for each sample. The samples were processed at the Paleoparasitology Laboratory Marie et Louis Pasteur University, France. Samples were examined under a light microscope Olympus BX-51 at the magnification of 100× and 400× using a Leica Application Suite V4.4.

### Morphological and morphometric analyses

The egg measurements were made as proposed by Borba and coauthors (2021a) noting length width, plug base length, plug base height, and shell thickness [24]. Eggshell surface morphotypes were separated on the basis of Borba and coauthors (2021b) [25], in four categories [smooth (S), punctuated (P), reticulated type I (RTI), and reticulated type II (RTII)] as follows: S type has no ornamentation on the egg surface as described by Zajac and Conboy in *Trichuris trichiura* (Linnaeus 1758) eggs [26]; P type present ornaments such as holes on eggshell, as little perforation all over the egg as described in *Eucoleus boehmi* (Supperer, 1953) by Conboy (2009) and Traversa et al. (2011) [26, 27]; RTI forms a network without an orientation as a grid described in *Eucoleus aerophilus* (Creplin, 1839) by Conboy [27]; different from RTII, which has a longitudinal orientation seems like a lot of small rays from one polar plug to another as described in *Aonchotheca putorii* (Rudolphi, 1819) by Zajac and Conboy (2012) [26].

### Statistical analysis

The species, deposited in two institutional collections, *Coleção Helmintológica do Instituto Oswaldo Cruz (CHIOC)*/Brazil and *Collection de Nématodes Zooparasites du Museum National d’Histoire Naturelle de Paris*/France, were used as references for the statistical analysis with eggs in archaeological samples. The statistical analysis had the objective of showing measures that are closer to one another within all the species throughout the database. Therefore, a discriminant analysis is applied to build a predictive model that generates a discriminant function on the basis of linear combinations to predict the best variable to distinguish the species within the groups [4]. From this point, hierarchical clustering using Gower distance and Ward minimum variance [28] is also applied to find proximities in measures, however, in a multivariate analysis using PAST 3.16 software [29].

### Artificial intelligence/machine learning approaches

An artificial intelligence/machine learning approach was applied in this study to propose/identify the species of specimens found, following Borba and coauthors (2021a) [24]. The database applied was from the two institutional collections, Oswaldo Cruz Institute Helminthological Collection (CHIOC/FIOCRUZ) and Collection of Zooparasite Nematodes of the Natural History Museum of Paris (MNHN), with 28 species characterized by morphology and morphometry, and when possible, host and geographical location. A decision tree (DT) was generated [24] with the information on egg morphology and morphometry (MM) and geographical location (GL) for European samples where the host is unknown, and a DT with all parameters, MM, host (H), and GL for Brazilian samples. For comparison, a reduced DT containing only the information of egg MM was included to extend the reach and maximize the possibilities of species.

## Results and discussion

**Fig. 1:**
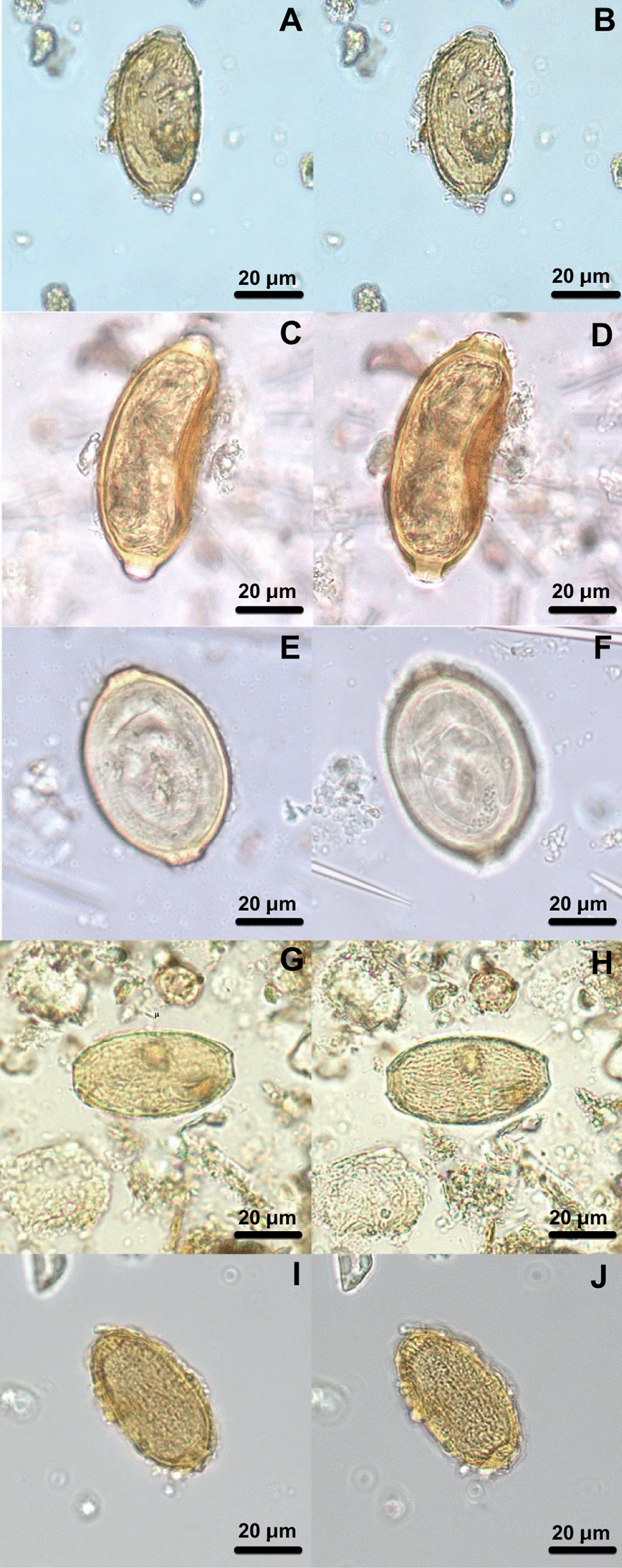
Capillariid eggs from New World samples. GGII-01 (**A**, **B**); GGII-15 (C–F); GGII-33 (G, H); GGII-51 (**I**, –). The first image of each egg focuses on egg structures (**A**, **C**, **E**, **G**, **I**), and the second image focuses on eggshell ornamentation (**B**, **D**, **F**, **H**, **J**). Light microscope at the magnification of 400×

**Fig. 2:**
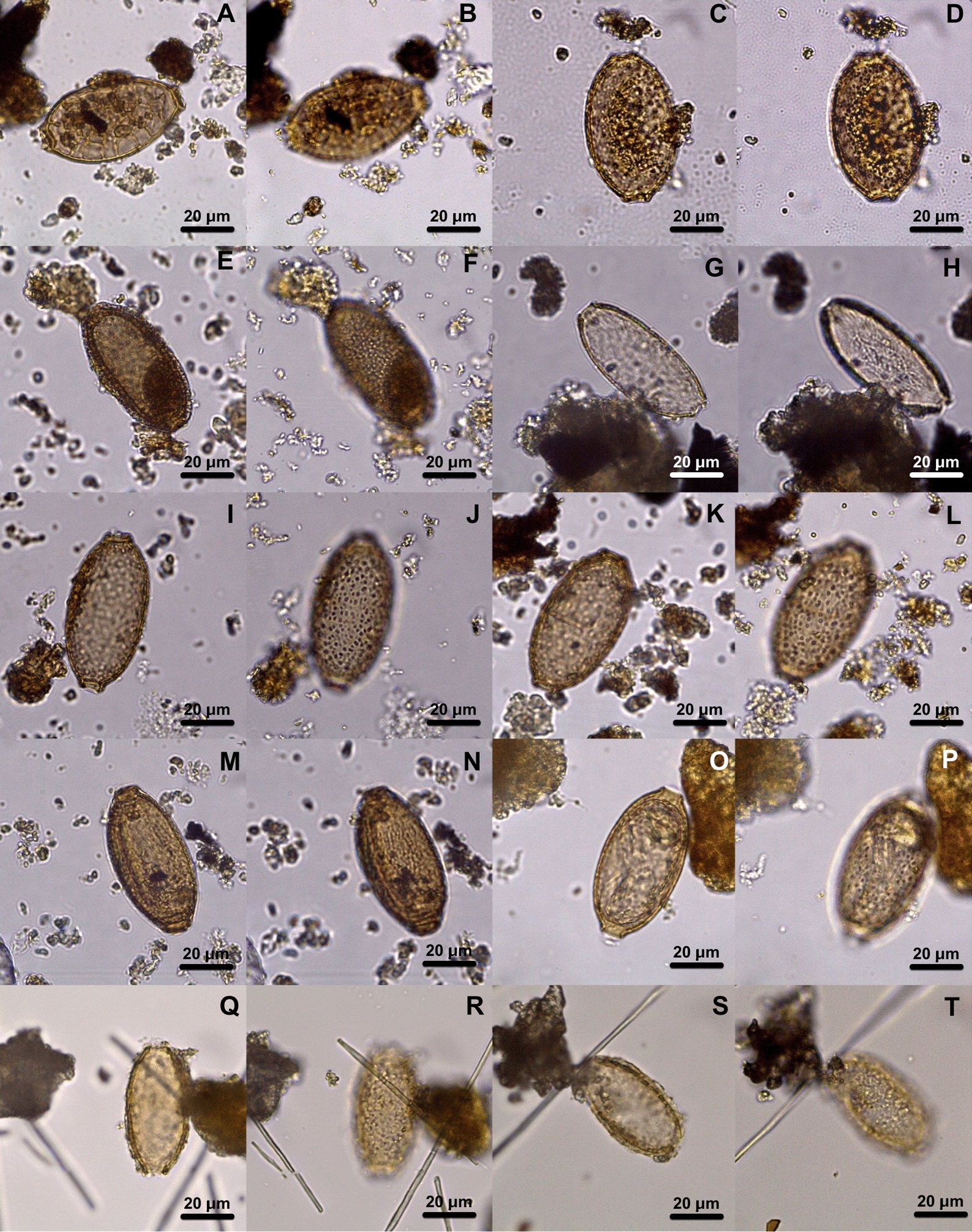
Capillariid eggs from Old World samples. AVA10-P17 (**A**, **B**); AVA10-P37 (**C**, **D**); AVA10-P21 (**E**, **F**); AVA10-P41 (**G**, **H**); AVA10-P28 (**I**, **J**); AVA10-P72 (**K**, **L**); AVA10-P73 (**M**, **N**); CAL-P530 (**O**, **P**); LRA201-03 (**Q**, **R**); LRA201-05 (**S**, **T**). The first image of each egg focuses on egg structures (**A**, **C**, **E**, **G**, **I**, **K**, **M**, **O**, **Q**, **S**), and the second image of each egg focuses on ornamentation (**B**, **D**, **F**, **H**, **J**, **L**, **N**, **P**, **R**, **T**). Light microscopy at the magnification of 400×

A total of 27 capillariid eggs from archaeological sites in the New World (Fig. 1) and the Old World (Fig. 2) were identified. These eggs were found in four positive samples in the archaeological sites GGII, two in LRA, one in CAL, and seven in AVA (Table 1, Figs. 1and2).

**Table 1:** Sample information and results of morphometric morphological analyses

Samples	No. of eggs	Length	Width	Plug L	Plug H	Eggshell	Morphotypes	Sample type
GGII-01	1	51.672	25.032	6.444	2.348	1.776	RTII	Coprolite
GGII-15	1	76.923	30.312	9.739	7.192	2.000	RTII	Coprolite
GGII-15	5	63.556–56.485	43.221–41.027	12.253–7.832	7.000	2.000	S	Coprolite
GGII-33	1	48.83	21.103	5.605	6.396	1.500	RTII	Coprolite
GGII-51	1	49.779	26.073	6.808	6.714	1.500	RTII	Coprolite
LRA201-03	1	47.771	24.917	7.985	3.287	1.832	RTI	Latrine
LRA201-05	1	46.265	26.484	5.811	2.106	3.476	P	Latrine
CAL-P530	2	57.073–58.404	31.19–31.971	8.648–9.299	3.088–3.156	1.14–1.542	P	Pits
AVA10-P17	1	50.744	28.275	8.071	4.558	3.926	RTI	Pits
AVA10-P28	1	58.39	29.687	8.665	3.676	0.938	P	Pits
AVA10-P37	2	56.282–55.491	34.497–34.771	9.197–9.367	1.935–2.037	0.938–1.606	P	Pits
AVA10-P73	1	57.79	28.858	9.952	3.861	1.832	RTII	Pits
AVA10-P72	2	56.9–58.963	31.292–30.941	9.726–9.538	3.294–2.653	2.661–1.192	P	Pits
AVA10-P41	1	57.678	25.491	8.444	2.005	1.107	RTII	Pits
AVA10-P21	6	56.336–51.321	27.381–31.376	9.162–7.470	4.864–3.97	3.361–4.31	P	Pits

Length and width of the total egg. Plug L: plug length, Plug H: plug height, Eggshell: eggshell thickness. Eggshell ornamentation morphotypes, S: smooth, P: punctuated, RTI: reticulated type I, RTII: reticulated type II. Measures in μm. The archaeological sites are GGII: *Gruta do Gentio II*, LRA: *La Rochelle Augustin*, CAL: *Calais ZAC de la Turquerie*, AVA: *Bourges Avaricum*

**Fig. 3:**
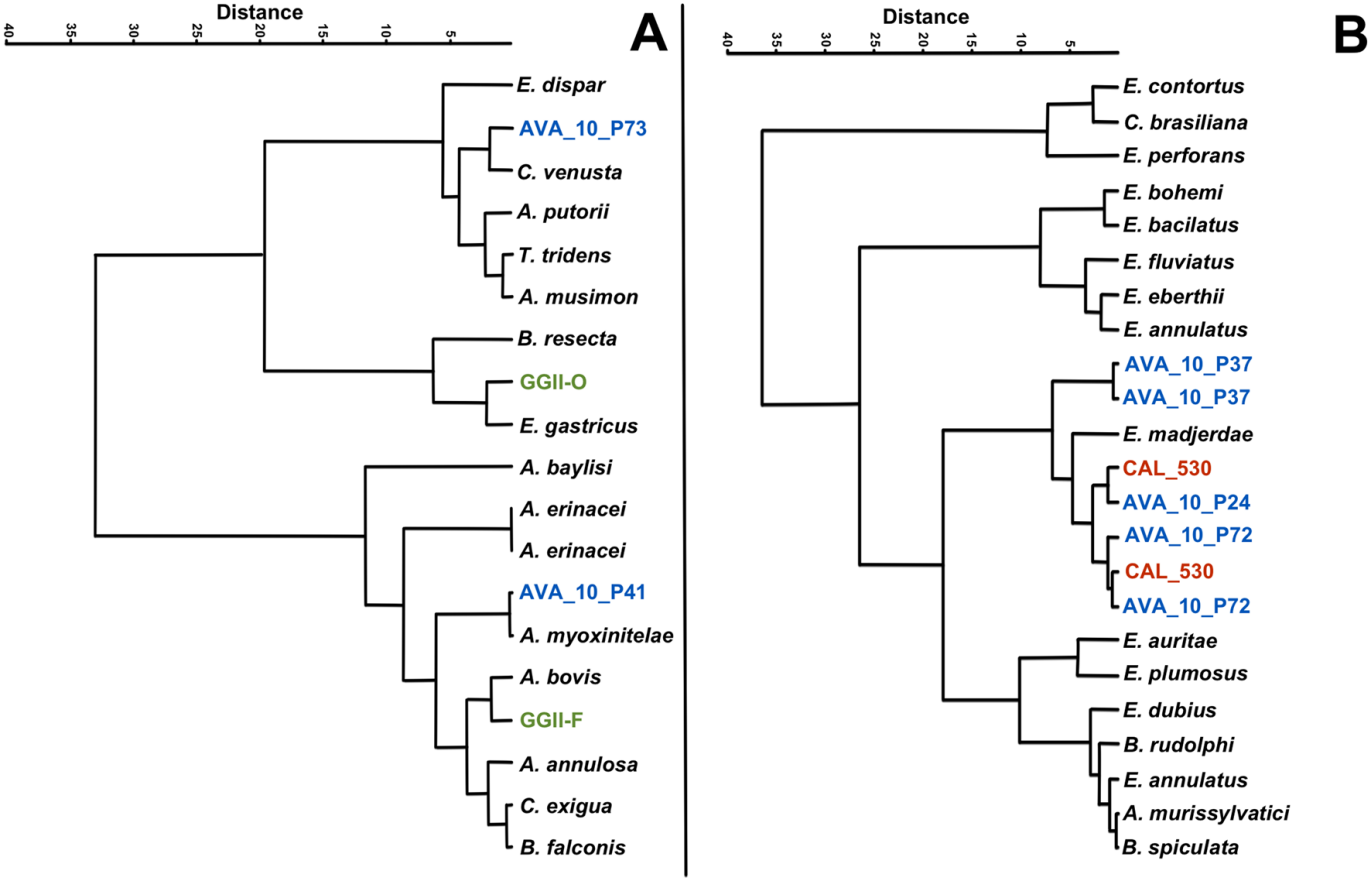
Hierarchical clustering of capillariid species by eggshell ornamentation using Gower distance and Ward minimum variance. Reticulated type II tree (**A**). Punctuated type tree (**B**). The archaeological sites are represented as Gruta do Gentio II (GGII), Calais ZAC de la Turquerie (CAL), Bourges Avaricum (AVA)

**Fig. 4:**
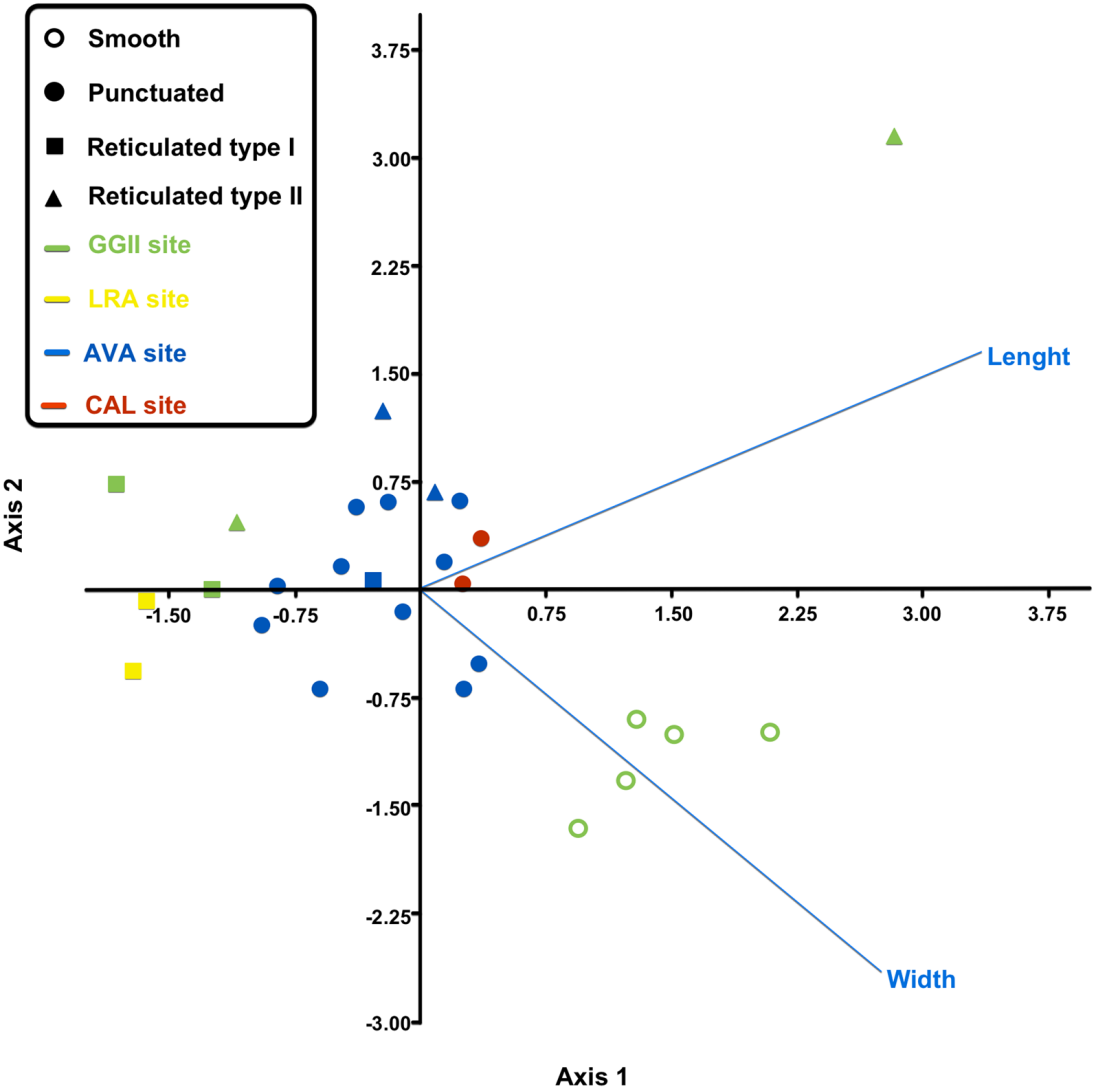
Discriminant analysis graphic with 27 capillariid eggs from archaeological sites distributed by length and width. The archaeological sites are represented as Gruta do Gentio II (GGII), La Rochelle Augustin (LRA), Calais ZAC de la Turquerie (CAL), Bourges Avaricum (AVA)

The discriminant analysis showed eggs by their length and width plotted on an *XY*-axis (Fig. 3). In addition, hierarchical clustering trees using Gower distance and Ward minimum variance were generated on the basis of the length and width measures of eggs per two ornamentation types of eggshells, reticulated type II (Fig. 4A), and punctuated (Fig. 4B).

Capillariids from the New World were recovered from coprolites in the present study. Sample GGII-01, identified with a *Panthera onca* origin [21], showed an egg with a RTII ornamentation (Fig. 1A, B). Eggs recovered from carnivores’ coprolites have to be considered as true infections or false parasitism, due to the ingestion of non-infective stage parasite eggs coming from their prey. The species that are usually found in felids are *C. hepaticum* (Bancroft, 1893) [30], *Eucoleus aerophilus* (Creplin, 1839) [31]*, Pearsonema feliscati* (Diesing, 1851) [32], and *P. plica* (Rudolphi, 1819) [33]. *Calodium hepaticum* parasites the liver and are present in feces only due to ingesting infected prey. GGII-01 has a particular eggshell structure different from *C. hepaticum* [4]. *Eucoleus aerophilus*, which parasites lungs, does not have the same eggshell ornamentation type observed in GGII-01 coprolite [34]. *Pearsonema feliscati* and *P. plica*, which usually infect the urinary tract, have punctuated and reticulated type I ornamentations, respectively. As none of the common capillariids found in felids matches in size or ornamentation, GGII-01 originates most likely from prey, which characterizes false parasitism. In the hierarchical tree of reticulated type II ornamentation (Fig. 4A), we can see GGII-01 eggs in a cluster with *Baruscapilaria falconis* (Goeze, 1782; Barus and Sergejeva, 1990) that infect birds of prey [35], *Capillaria exigua* (Dujardin, 1845), and *A. annulosa* (Dujardin, 1845) that infect rodents and *A. bovis* (Schnyder, 1906) that infect bovids [36]. All these species are not specific to felids but can parasite a potential prey. The identification by the decision tree generated by AI/ML using all parameters (MM + H + GL) resulted in *Capillaria venusta* [37] when considered a bird prey, or in *Capillaria exigua* if the prey was a mammal, which corroborates in this case with the hierarchical clustering. Although *Capillaria exigua* is a parasite of hedgehogs distributed in Europe and not in the Americas, the characteristics and measurements are compatible. As the reference dataset was used as an initial approach, some misdiagnoses were expected since only 28 species were available with all the parameters accessed, from more than 300 species of capillariids described worldwide [5].

Coprolite GGII-15 from *Didelphis albiventris* [21] presents two different capillariid morphotypes. One bigger (GGII-15-01) measuring 76.92 × 30.31 μm, with a discreet RTII ornament (Fig. 1C, D), and a second (GGII-15-02) represented by five rounder and smaller eggs (63.55–56.48 × 43.22–41.02 μm) with no ornament (Fig. 1E, F). Spratt [38] described 14 species of capillariid parasitizing marsupials, 4 of them presented reticulated or reticulated-like eggshell ornaments: *Eucoleus gastricus* (Baylis, 1926) (65–79 × 25–34), *E. fluvidus* (Spratt, 2006) (65–72 × 26–31 μm), *E. plumosus* (Spratt, 2006) (60–65 × 21–25 μm), and *E. pseudoplumosus* (Spratt, 2006) (50–56 × 21–25 μm). Despite the measurements of GGII-15–01 matching with *E. gastricus*, the egg morphology is different from the one found in archaeological samples from France during the First World War [28]. In the cluster analysis, it was related to *E. gastricus* (Fig. 1A, B). Although it usually parasites rodents, *E. gastricus* has been described in marsupials from Australia [38]. It could also be another species not yet described in marsupials in Brazil. Since all the eggs similar to the one found in GGII-15 are from *Eucoleus* spp., we make the hypothesis that it belongs to the *Eucoleus* genus. In AI/ML analysis, using the DT with all parameters (MM + H + GL), it is identified as *A. myoxinitelae*, a species that infects rodents. The DT with only MM matches with *Baruscapillaria resecta* (Dujardin, 1845), a parasite of birds. The species *E. gastricus* was not present in the AI dataset, so it was not possible to corroborate with the other diagnosis using only literature. We also have to consider that the width measurement in the egg found is impaired and can give a false species definition in this case. *Baruscapillaria resecta* and *A. myoxinitelae* are found in Europe [39] and North America [40], while *E. gastricus* is found worldwide [41], therefore, it is more likely to be this parasite species. The reason GGII-15–01 was identified as *A. myoxinitelae* even when using all the parameters is that specimens deposited in the Institutional Collections do not have an assigned geographical location. Opossums have a diverse diet, with birds and small mammals as possible food resources [42], thus the egg could come from any of these species.

The measurements of the second GGII-15 morphotype are not compatible with the only capillariid without ornamentation known in the dataset that generated the DT, *Aonchotheca pulchra* (Freitas, 1934). Other species of the same genus have similar eggshells, such as *Aonchotheca italica* (Ricci, 1949) and *Aonchotecha eubursata* (Skarbilovitsch, 1946), all parasites of bats [43]. The other species described in the host infraclass do not have the same egg morphology, however, we have to consider other Trichocephalida species as *Trichosomoides* genus, which parasites the urinary bladder of rodents and has compatible egg morphometry and similar morphology [44, 45].

GGII-33 (Fig. 1G, H) and GGII-51 (Fig. 1I, J) found in *Bos taurus* samples [20], have a morphology compatible with *Aonchotheca bovis* (Schnyder, 1906), a parasite specific to bovid. However, the measures are a bit smaller than those designated in the literature (50–54 × 24–31 μm) [36]. The bovid host, whose parasite species were identified, *Cervus elaphus* (Artiodactyla) [36], is different from the *B. taurus* genetic identification of the GGII sample. A distinct host could produce a plasticity effect in the parasite morphology, as described in other species, such as *A. putorii* (Rudolphi, 1819) [46]. Another matter is that the layer where the coprolite was found is dated as pre-Colombian, a period when there was no *Bos taurus* in the New World. It could be explained as a post-Colombian perturbance of the layers and/or a recent feces deposit between excavation expeditions [20]. When it was applied, the DT with MM + GL matches with *C. venusta*, and with only MM, matches with *C. exigua*, both with similar ornamentation with the eggs found, but not compatible with the expected host.

The Old World samples analyzed do not have a defined host since pits and latrines contain a lot of organic material, including human and animal feces [35, 47]. The eggs of the AVA10-P21 (56.336–51.321 × 27.381–31.376 µm) sample have a thick shell with dense punctuated ornamentation, with radial visualization on a transversal view, characteristics that are very particular to those of *C. hepaticum* [48]. Obviously, there could be other species with similar eggshell features as reported by other authors for *Calodium soricicola* (Yokogawa and Nishigori, 1924) [43]. This species can parasite the liver of a mammalian host, including humans, but visualization in feces is only with the ingestion of contaminated viscera. The AI/ML approach matches *C. hepaticum* as expected.

One egg morphotype was seen in three different samples, AVA10-P28 (58.39 × 29.687 μm), AVA10-P72 (56.9–58.96 × 31.29–30.94 μm), and CAL-530 (57.07–58.40 × 31.19–31.97 μm). The ornamentation is punctuated-like, with large holes over eggshells, which gives the appearance of a reticulated type (Fig. 2I–L, O, P). The species deposited in the Institutional Collections used as references, with similar ornamentation, are *Eucoleus dubius* (Travassos, 1917), *E. eberthii* (Freitas e Lent, 1935), and *E. madjerdae* (Bernard, 1964) [49], which parasitize *Attila cinereus* (Passeriformes), *Metachirops opossum* (Didelphimorphia), and *Mus musculus* (Rodentia), respectively. The morphotype can be seen in proximity in the hierarchical tree with *E. madjerdae*, which have egg morphologies similar to the ones found in the samples. The result is corroborated by AI/ML analysis, which identifies *E. madjerdae* when the DT with only MM parameters was applied, however, when DT with MM + GL parameters was applied it resulted in *A. murissylvatici*. Both capillariids have similar morphology and morphometry, although *A. murissylvatici* is described in France. *Eucoleus boehmi*, which has similar egg morphology, was not included in AI/ML dataset analysis, as it was not deposited in the institutional collections.

The sample AVA10-P37 (56.28–55.49 × 34.49–34.77 μm) had an egg with similar ornamentation, morphology, and eggshell thickness to the other three samples mentioned above, but with a bigger width and smaller length. However, the proximity with *E. madjerdae* in the hierarchical tree was suggested as *B. spiculata* (Freitas, 1933) by AI/ML procedure. *Eucoleus madjerdae* was deposited in MNHN collection and described as a parasite of rodents collected in Tunisia, as *E. boehmi* parasites feline and canids in Europe [50], which is a plausible finding in this samples as well.

In samples, AVA10-P41 (57.67 × 25.49 μm) and AVA10-P73 (57.79 × 28.85 μm) eggs were found with RTII ornaments, longitudinal rays, such as a striate surface (Fig. 2G, H, M, N). Although these two eggs have the same ornamentation pattern and measurements (Table 1), their morphology and eggshell thickness are not compatible, thus we classified them as different species morphotypes. Both have a close distance between different species in the hierarchical clustering analysis. AVA10-P41, with *A. myoxinitelae* (Diesing, 1851), parasites the liver of rodents and boar in France [51]. AVA10-P73, with *Capillaria venusta* (Freitas e Mendonca, 1958), infects the intestine of neotropical birds and Piciformes, which are globally distributed [52]. The morphologies and the morphometries match each species’ record, and both identifications corroborate with AI/ML results. Thus, we can suggest a circulation of these animals that are parasitized in these areas, or have cohabitation and contact with the human population in this region.

Capillariids from samples LRA201-03 and LRA201-05 are the smallest eggs found in this study (Fig. 2Q–T). They both have dense punctuations that look like a RTI ornamentation, as a grid. It is noted that the outer shell is damaged, which can impair the visualization of ornamentation. Both are very similar in morphology and size, but the ornaments are slightly different; LRA201-03 has a bigger punctuation unit than LRA201-05. Considering RTI and P ornamentations, respectively, the AI/ML analysis identified *Capillaria collaris* (Linstow, 1873) and *Eucoleus annulatus* (Molin, 1858), both parasites of birds and found in Europe [53]. The morphology is not very similar, which indicates the necessity for more characterization of species in the group (Table 2).

**Table 2:** Results of capillariid species identification based on discriminant analysis, hierarchical clustering, and artificial intelligence/machine learning

Samples	Literature	Discriminant analysis	Hierarchical clustering analysis	Artificial intelligence/machine learning analysis		
				DT MM + GL + H	DT MM	DT MM + GL
GGII-01	–	*A. erinacei* *C. exigua* *A. annulosa*	*B. falconis* *C. exigua* *A. annulosa* *A. bovis*	*C. venusta* *C. exigua*	–	–
GGII-15-01	*Eucoleus* sp.	*E. gastricus*	*E. gastricus*	*A. myoxinitelae*	*B. resecta*	–
GGII-15-02	*A. italica* *A. eubursata*			*A. pulchra*		
GGII-33	*A. bovis*	–	–	–	*C. exigua*	*C. venusta*
GGII-51	*A. bovis*	–	–	–	*C. exigua*	*C. venusta*
LRA201-03	–	–	–	NA	–	*C. collaris*
LRA201-05	–	–	–	NA	–	*E. annulatus*
CAL-P530	–	*-*	*E. madjerdaei*	NA	*E. madjerdae*	*A. murissylvatici*
AVA10-P17	–	–	–	NA	–	–
AVA10-P28	–	–	*E. madjerdae*	NA	*E. madjerdae*	*A. murissylvatici*
AVA10-P37	*E. boehmi*	–	*E. madjerdae*	NA	*B. spiculata*	*B. spiculata*
AVA10-P73	–	–	*C. venusta*	NA	*C. venusta*	*C. venusta*
AVA10-P72	–	–	*E. madjerdae*	NA	*E. madjerdae*	*A. murissylvatici*
AVA10-P41	–	–	*A. myoxinitelae*	NA	*A. myoxinitelae*	*A. myoxinitelae*
AVA10-P21	*C. soricicola* *C. hepaticum*	–	–	NA	*C. hepaticum*	*C. hepaticum*

The archaeological sites are GGII: *Gruta do Gentio II*, LRA: *La Rochelle Augustin*, CAL: *Calais ZAC de la Turquerie*, AVA: *Bourges Avaricum*

DT: decision tree, parameters considering MM: morphological and morphometric data, GL: geographical location, H: host. Genera named are *A: Aonchotheca*, *C: Capillaria*, *E: Eucoleus B: Baruscapillaria*. NA: not applicable since European samples do not have host information

The egg found in sample AVA10-P17 has a particular structure (Fig. 2A, B). Apparently, the outer shell is S-type and there is a large grid in its interior. A similar description was not found in the institutional collections nor in the literature. For this reason, no AI/ML approach was applied. Although it can be described as RTI ornamentation, it is not similar to any egg characterized in the study.

The study corroborates that the application of the AI/ML methodology in samples from the New World with host definition gives a possibility of deeper discussion about taxonomic egg identification, as previously demonstrated [5]. The ML/AI method already proposed revealed that when GL and H parameters were included in addition to the MM parameter, the reliability of the DT was higher with all algorithms used [24], as is the case of samples of the New World. While those from the Old World, which were from latrines and pits, made the investigation, they were only based on egg morphology and eggshell ornaments (MM), and it was difficult since parasites could be from any host, animal or human. The limited data about the host/producer of samples could be overtaken using genetic analysis to determine the origin of the sample in the case of isolated coprolites [21], which is more difficult when working with latrine samples. The human or other vertebrate animal origin of the sample could be achieved by standard DNA barcoding analysis, with a robust dataset available at GenBank. This is not true for helminths, especially, nor for Capillariidae species, which have little or absent genetic information available. In addition to the limited information on the ancient parasite eggs to perform a species definition or discuss an approximation, we are aware that the ML/AI approach has some restrictions, especially regarding the reference dataset. Despite it being a solid representation of capillariids available in two important institutional biological collections, CHIOC/FIOCRUZ and *Collection de Nématodes Zooparasites*/MNHN, it contains only 28 species and 8 genera out of more than 300 species and 25 genera capillariid described [24]. Another question is whether the metadata associated with the reference species with multiple hosts or geographical origins could be wrongly addressed by the system as a discrepant character [24]. For all these limitations, we agreed that the increment of the Capillariidae dataset, including new curated information from other institutional helminth collections, will allow for a better taxonomic definition using ML/AI.

## Conclusions

The analyses conducted in the present study validated that eggshell surface ornamentation is the most relevant character for the taxonomic definition of capillariids. In the literature, there is not much image of the capillariid eggshell surface, which precludes a proper identification of species. The study shows the importance of egg description and characterization to create a source of comparison for species identification. The study is unprecedented in applying the deposits of curated species from institutional biological collections and technologies of artificial intelligence in the paleoparasitological analysis for identifying parasitic structures from archaeological sites and emphasizes the importance of biological collections as a taxonomic reference, not only of adult forms as traditionally used, but also in parasite eggs, which are the structures most often found in paleoparasitological analyzes and parasitological diagnosis. The results provide new data for capillariid species definition, allowing for a better understanding of the relationship between parasites and human or animal hosts in the past.

## Data Availability

Data are provided within the manuscript.
